# Real-Time RT-PCR for the Detection of Lyssavirus Species

**DOI:** 10.1155/2014/476091

**Published:** 2014-10-16

**Authors:** A. Deubelbeiss, M.-L. Zahno, M. Zanoni, D. Bruegger, R. Zanoni

**Affiliations:** Institute of Virology and Immunology, 3012 Berne, Switzerland

## Abstract

The causative agents of rabies are single-stranded, negative-sense RNA viruses in the genus *Lyssavirus* of Rhabdoviridae, consisting of twelve classified and three as yet unclassified species including classical rabies virus (RABV). Highly neurotropic RABV causes rapidly progressive encephalomyelitis with nearly invariable fatal outcome. Rapid and reliable diagnosis of rabies is highly relevant for public and veterinary health. Due to growing variety of the genus *Lyssavirus* observed, the development of suitable molecular assays for diagnosis and differentiation is challenging. This work focused on the establishment of a suitable real-time RT-PCR technique for rabies diagnosis as a complement to fluorescent antibody test and rabies tissue culture infection test as gold standard for diagnosis and confirmation. The real-time RT-PCR was adapted with the goal to detect the whole spectrum of lyssavirus species, for nine of which synthesized DNA fragments were used. For the detection of species, seven probes were developed. Serial dilutions of the rabies virus strain CVS-11 showed a 100-fold higher sensitivity of real-time PCR compared to heminested RT-PCR. Using a panel of thirty-one lyssaviruses representing four species, the suitability of the protocol could be shown. Phylogenetic analysis of the sequences obtained by heminested PCR allowed correct classification of all viruses used.

## 1. Introduction

Rabies diagnosis is based on fluorescent antibody testing (FAT) in brain smears and inoculation of brain suspension either in mouse neuroblastoma cell cultures or intracerebrally in mice as confirmatory assays with high sensitivity and specificity for postmortem diagnosis [1–3]. These techniques are well suited for rapid and reliable routine diagnosis, if brain material is available. For other diagnostic specimens sampled intra vitam in case of clinical suspicion, for example, saliva, cerebrospinal fluid, or skin biopsies, more suited molecular techniques with excellent sensitivity have been developed and validated, mostly targeting the conserved nucleoprotein gene of the lyssavirus genome, for example, [4–10]. Among these, there are protocols both for classical RT-PCR and for real-time RT-PCR, which adds speed, efficiency, contamination safety, and reliability to the technique combined with the potential to quantify the viral load [11, 12]. A major advantage offered by these molecular techniques is the characterisation of viral isolates by sequencing the given amplification products followed by phylogenetic or phylogeographic analysis [13–15]. The greatest challenge for these powerful novel techniques is the ever growing spectrum of known lyssavirus species/genotypes (GT), probably all of which having the potential to cause animal and human rabies fatalities [16, 17]. So far, 12 lyssavirus species and three as yet unclassified species have been identified [17]. The development of classical simple or (hemi)nested RT-PCR methods with particular emphasis on the diagnosis of a broad spectrum of lyssaviruses species has been published in a number of studies [18–23]. Also several real-time RT-PCR protocols suited for the broad detection or differentiation of several genotypes have been developed [9, 24–28]. The work of Wakeley et al. [25] is of particular interest for the rabies epidemiology in Europe concerning the European bat lyssaviruses types 1 and 2 [29–32] apart from classical rabies [33, 34]. This assay uses genotype (GT) specific probes and fluorophore signals for direct differentiation of GT1, GT5, and GT6 in a single-tube reaction. The protocol proposed by Nadin-Davis et al. [8] is highly suited for the detection of a broad range of classical rabies viruses.

The goal of this work was, based on a comprehensive review of the rich literature on the theme and adapting thereof, to develop, establish, and validate classical and real-time RT-PCR protocols for (intravitam) diagnosis of rabies and molecular-epidemiological characterisation of viral strains with the main emphasis on the growing number of known species/genotypes.

## 2. Materials and Methods

### 2.1. Samples

#### 2.1.1. Cell Culture Supernatant of Viral Strains

All operations with potentially infectious material apart from centrifugation in closed tubes were performed in a class II biological safety cabinet. Frozen cell culture supernatants of rabies virus strains propagated previously on BHK-21 cells (baby hamster kidney cells, ATCC, Manassas, USA) were thawed, centrifuged at 2,000 g for 10 min at 4°C, and filtrated using the Millex-HA 450 nm Filter (Millipore, Cork, Ireland).

#### 2.1.2. Brain Suspension

Approximately 1 g of cerebellum, medulla oblongata, and hippocampus from unfixed, freshly obtained brain material was homogenized to a 20% brain suspension using a mortar and pestle, after addition of approximately 1 g of quartz sand (Merck KGaA, Darmstadt, Germany) and 5 mL of Modified Eagle Medium with Earle's salts with 2.2 g/L NaHCO_3_ (Bioswisstec AG, Schaffhausen, Switzerland) supplemented with 5% penicillin 10,000 IU/mL (Bioswisstec AG, Schaffhausen, Switzerland) and 20% foetal calf serum (FCS; PAA Laboratories GmbH, Pasching, Austria). The suspension was then decanted into a 5 mL tube (5 mL Polystyrene Round-Bottom Tube; BD Biosciences, Erembodegem, Belgium) to let it sediment for 1 hour at 4°C and subsequently centrifuged at 1,400 g for 10 min and filtrated as above. Frozen brain suspensions from former mouse inoculation tests [39] were handled like frozen cell culture supernatant.

#### 2.1.3. Saliva and Oral Swabs

500 *μ*L of RNA Storage Solution (Ambion, Foster City, USA) was added to 100–200 *μ*L of fresh or previously frozen saliva samples or swabs, which were subsequently vortexed (Vortex Genie 2, TEWIS Laborbedarf AG, Berne, Switzerland) for 1 min and centrifuged in a Biofuge Pico (Heraeus Holding GmbH, Hanau, Germany) at 5,000 rpm for 10 min.

#### 2.1.4. Cerebrospinal Fluid

Cerebrospinal fluid (CSF) samples were diluted 1 : 2 to 1 : 4 with RNA Storage Solution (Ambion, Foster City, USA).

#### 2.1.5. Skin Biopsies

Skin biopsies were shaved, cut into small pieces with a sterile pair of scissors, and further processed as described for brain suspension.

### 2.2. Viral Strains and Propagation in Cell Culture

A panel of 31 lyssaviruses (including 4 species) was used to test the diagnostic performance of our PCR (Table 1). Stocks of viral strains were produced in neuroblastoma cells (MNA 42/13) as described for the rabies tissue culture infection test (RTCIT, described below). The maintenance medium used for the first passage was Dulbecco's MEM supplemented with 0.5% neomycin 50 IU/mL (Bioswisstec AG, Schaffhausen, Switzerland), 5% tryptose phosphate (BioConcept Ltd. Amimed, Allschwil, Switzerland), 1% nonessential amino acids (Bioswisstec AG, Schaffhausen, Switzerland), 3% foetal calf serum, and 1% L-Glutamine 200 mM (Bioswisstec AG, Schaffhausen, Switzerland) to which 1% Diethylaminoethyl-Dextran (DEAE-Dextran; Sigma-Aldrich Corporation, St. Louis, USA) was added. Dulbecco's MEM with 10% foetal calf serum was used for the 3 consecutive passages. Staining of the cells was performed after each passage using Rabies DFA Reagent (Millipore, Livingston, UK) as a conjugate.

### 2.3. FAT and RTCIT

The standard fluorescent antibody test (FAT) was performed with brain tissue samples as previously described [40]. Rabies tissue culture infection test (RTCIT) using four consecutive passages on murine neuroblastoma cells [41–43] was applied to clinical specimens like brain specimens, liquor, saliva, or skin biopsies.

### 2.4. Primers, Probes, and Synthetic DNA

Primers, probes, and synthetic DNA were obtained from Microsynth (Microsynth GmbH, Balgach, Switzerland). The location of suitable N-directed primers and probes for heminested RT-PCR and real-time RT-PCR, respectively, was chosen and evaluated based on published work [8, 18, 22, 25]. Using multiple sequence alignment (ClustalX 2.0.3 program [44–46]) of the N gene region of the available lyssavirus species RABV (33 sequences), LBV (4 sequences), MOKV (5 sequences), DUVV (4 sequences), EBLV-1 (17 sequences), EBLV-2 (14 sequences), ABLV, Aravan, Khujand, Irkut, and West Caucasian bat (each one) from GenBank (National Center for Biotechnology Information and National Library of Medicine, Rockville Pike, USA), variable positions of primers and probes were adjusted with wobble positions for a potentially broader match. Alternatively, primers with single wobbles or substitutions were mixed. Several probes for real-time RT-PCR for broadening the spectrum of detectable species were designed in this work (Table 2). As internal control for conventional RT-PCR, amplification of GAPDH (glyceraldehyde 3-phosphate dehydrogenase) was used [37]. As internal inhibition control for real-time RT-PCR, primers and probes for the amplification of Sendai virus (ATCC, Manassas, USA [38]), which was added to the samples before RNA isolation, were used (Table 2).

Synthetic DNA with a length of 125 bases encompassing positions 48–172 according to the Pasteur virus genome (X03673) of the following lyssavirus species were obtained from Microsynth: Aravan virus (ARAV) EF614259, Khujand virus (KHUV) EF614261, Bokeloh bat lyssavirus (BBLV) JF311903, Australian bat lyssavirus (ABLV) AF418014, Irkut virus (IRKV) EF614260, Lagos bat virus (LBV) EU293110, Mokola virus (MOKV) 293117, Shimoni bat virus (SHIV) GU170201, and West Caucasian bat virus (WCBV) EF614258.

### 2.5. RNA Extraction

RNA was isolated from 140 *μ*L of sample supernatant using the QIAamp Viral RNA Mini Kit (QIAGEN, Germantown, USA) according to the manufacturer's instructions, except the usage of RNA Storage Solution (Ambion, Foster City, USA) for elution. Extracted RNA was stored at −20°C until use.

### 2.6. Heminested RT-PCR

Heminested RT-PCR was performed as previously described [22] using the OneStep RT-PCR Kit. Briefly, for the first round 3 *μ*L of extracted RNA was amplified in the RT-PCR mix prepared according to manufacturer's instructions, supplemented with 0.6 *μ*M of each primer (JW12-F, JW6AS1-R1, and JW6AS2-R1; Table 2) and 5 U RNase inhibitor (RNasin Plus, 40 U/*μ*L; Promega, Madison, USA), with the following cycling conditions: 50°C for 30 min and 95° for 15 min for reverse transcription and subsequent activation of the polymerase, followed by 10 cycles of 95°C for 20 s, 60°C for 30 s (1°C decrease/cycle), followed by 72°C for 30 s and 35 cycles of 95°C for 20 s, 52°C for 30 s, followed by 72°C for 30 s. For the second round, the Taq DNA Polymerase Kit (QIAGEN, Germantown, USA) was used with 2 *μ*L of the first round product, 2.5 *μ*L 10x PCR buffer, 1.2 mM final dNTP concentration (Invitrogen, Foster City, USA), 0.4 *μ*M of heminested primers (JW12-F, JW10AS1-R2, and JW10AS2-R2; Table 2), 0.5 U of Taq DNA polymerase, and 19.35 *μ*L Nuclease Free Water (Ambion, Foster City, USA). Cycling conditions were as follows: 95°C for 5 min followed by 35 cycles of 94°C for 20 s, 52°C for 20 s, followed by 72°C for 30 s. Amplifications were performed in a 2720 Thermal Cycler. Internal controls using GAPDH primers, as described in the one-round PCR, were run in parallel in the first round.

### 2.7. Gel Electrophoresis for Sequencing

Gel electrophoresis was performed using a 1.5% TAE agarose gel (agarose LE, analytical grade; Promega, Madison, USA) stained with ethidium bromide (Eurobio, Courtaboeuf, France). For sequencing, the desired band was excised under UV light [47].

### 2.8. Real-Time RT-PCR (NDWD)

Real-time RT-PCR (NDWD) adapted from Nadin-Davis et al. and Wakeley et al. [8, 25] was performed using the QuantiTect Probe RT-PCR Kit (QIAGEN, Germantown, USA). The 25 *μ*L reaction volume consisted of 9.5 *μ*L RNase-free water, or 9 *μ*L in the multiplex mix, 12.5 *μ*L 2x QuantiTect RT-PCR Master Mix, 0.25 *μ*L of the RABVD1 forward and reverse primer (0.4 *μ*M final concentration each), 0.25 *μ*L of the probes RABVD1, LysGT5, and LysGT6 together in the multiplex mix or 0.25 *μ*L of the probes ivvWCBV-P, ivvMok-P, ivvABLV2n-P, ivvLBV-P, ivvKhujand-P, and ivvSBLV2-P (Table 2) alone in a mix (all 0.2 *μ*M final concentration), 0.25 *μ*L of QuantiTect RT Mix, and 2 *μ*L of sample RNA. Additionally, for the internal Sendai virus control, 5 *μ*L of sample RNA was added to a mix containing 6.625 *μ*L of RNase-free water, 12.5 *μ*L 2x QuantiTect RT-PCR Master Mix, 0.625 *μ*L of Sendai forward primer, reverse primer, and probe mix (Sendai-F, Sendai-R, and Sendai-P, 8 *μ*M; Table 2), and 0.25 *μ*L of QuantiTect RT Mix. The reactions were carried out in MicroAmp Fast Optical 96-Well Reaction Plates with Barcode 0.1 mL (Applied Biosystems, Foster City, USA) in a 7500 Fast Real-Time PCR System v1.3.1 (Applied Biosystems, Foster City, USA) using the following cycling conditions: 1 cycle of 50°C for 30 min and 95°C for 15 min followed by 45 cycles of 95°C for 15 sec and 50°C for 1 min. Amplification curve analysis was performed using the 7500 Software v2.0.6 (Applied Biosystems, Foster City, USA).

### 2.9. Cloning for Analytical Sensitivity Analysis

For cloning, a segment of viral genome covering a part of the N gene (positions 1–933 according to the Pasteur virus genome accession number X03673) of CVS-11 was generated using primers designed for this purpose (TWclon-F and TWclon-R, Table 2). Amplification was performed using the OneStep RT-PCR Kit with the master mix consisting of 29.25 *μ*L of RNase-free water, 10 *μ*L 5x QIAGEN OneStep RT-PCR buffer, 0.4 mM of each dNTP, 0.6 *μ*M of the cloning primers, 10 U RNasin (40 U/*μ*L), and 2 *μ*L of QIAGEN OneStep RT-PCR Enzyme mixed with 5 *μ*L of extracted RNA to a total volume of 50 *μ*L. Amplifications were performed in a Veriti 96-Well Thermal Cycler (Applied Biosystems, Foster City, USA) using the following cycling conditions: 30 min at 50°C, followed by 15 min at 95°C and 40 repetitive cycles of 1 min at 94°C, 1 min with a temperature gradient from 56°C to 46°C in steps of 2°C, and 1 min at 72°C. Elongation at 72°C was extended for 10 additional minutes in the last cycle. After gel electrophoresis excised PCR fragments with a length of 933 bp were eluted with the QIAquick Gel Extraction Kit (QIAGEN, Germantown, USA) and cloned into the pCR-II-TOPO vector using the TOPO TA Cloning Kit (Invitrogen, Foster City, USA) according to the manufacturer's instructions.

### 2.10. Sequencing

Sequencing reactions were performed by Microsynth AG, Balgach, Switzerland. For this purpose, reaction mixtures containing 22.5 ng DNA per 100 bp, 30 pmol of each primer used for the amplification (Table 2), and DEPC treated water (Ambion, Foster City, USA) to a final volume of 15 *μ*L were prepared. Sequences were edited using the SeqMan II v5.01 Software (DNASTAR, Madison, USA) and subsequently evaluated using the Clone Manager 9 software (Scientific & Educational Software, Cary, USA).

### 2.11. Phylogenetic Reconstruction

The 543 bp nucleotide sequences of the nucleoprotein gene obtained from the heminested PCR reaction, together with additional sequences retrieved from GenBank, were saved in Fasta file format. The sequences were then aligned using the ClustalX 2.0.3 Software and the GeneDoc Software v2.5.0 [48]. Phylogenetic trees were constructed with the MEGA5 software using Kimura 2-parameter distances [35].

## 3. Results

### 3.1. Cell Culture

Thirty-one lyssaviruses (19x RABV, 7x EBLV-1, 4x EBLV-2, and 1x DUVV) were analysed in RTCIT. All isolates were viable in murine neuroblastoma cells and could be visualized using Rabies DFA Reagent as a conjugate.

### 3.2. Sensitivity of PCR Methods

**Figure 1:**
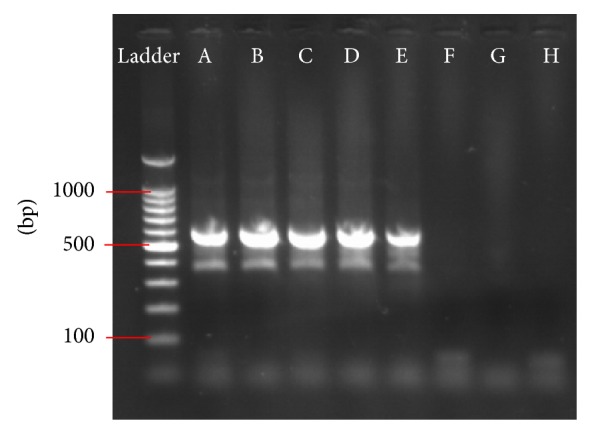
Amplification of a CVS-11 serial dilution using hnRT-PCR. Ethidium bromide stained gel after amplification of the CVS-11 strain using dilutions from 10^−1^ to 10^−8^ (A–H). The amplification product of 582 bp is clearly visible up to a dilution of 10^−5^ (lane E). Ladder = standard for determination of amplification product size.

For the comparison with infectious titres, RNA of the classical rabies virus strain CVS-11 was extracted from cell culture supernatants (BHK-21 cells or neuroblastoma cells MNA 42/13) and serially diluted from 10^−1^ to 10^−8^. The infectious titre was determined according to the Spearman-Karber formula [49, 50] in fluorescent focus forming doses 50% (FFD_50_). In the heminested RT-PCR (hnRT-PCR), the amplification product of 582 bp was visible up to a dilution of 10^−5^ corresponding to a detection limit of 0.4 FFD_50_ (10^−0.4^ FFD_50_; Figure 1). In the real-time RT-PCR (NDWD) the serial dilution was performed in triplicate. The limit of detection was defined as the last dilution at which at least 2 out of 3 replicates were positive. Using this definition, CVS-11 was detectable up to a dilution of 10^−7^ (once at a dilution of 10^−8^). The amplification plot showed regular intervals of the curves with CT-values from 18.1 (dilution 10^−1^) to 40.0 (dilution 10^−7^). Relating to infectious titre real-time RT-PCR reached a detection limit of 0.003 FFD_50_ (10^−2.6^) for CVS-11.

**Figure 2:**
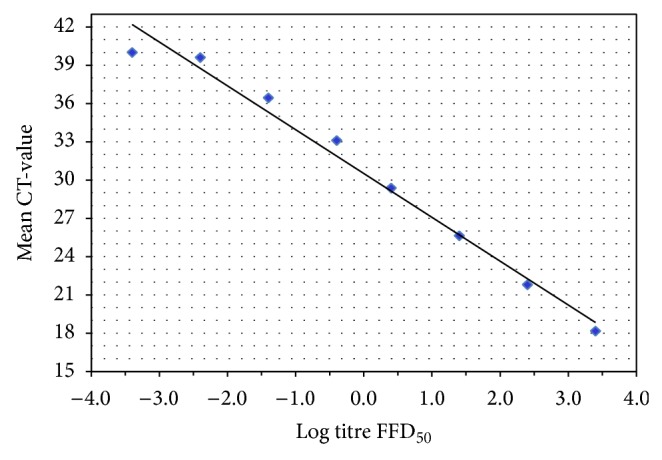
Efficiency of the real-time RT-PCR (NDWD). The linear regression of the CT-values (ordinate) and the titres of serially diluted CVS-11 (abscissa) exhibited a slope coefficient of −3.46 corresponding to an efficiency of 94.5% (*E* = 10^(−1/slope  coefficient)^ − 1).

The efficiency of the real-time RT-PCR (NDWD) was determined using linear regression of the CT-values and the titres of the samples (Figure 2). On the basis of the slope coefficient, the efficiency can be inversely derived as *E* = 10^(−1/slope  coefficient)^ − 1. The efficiency for CVS-11 was 94.5% with a slope coefficient of −3.46.

### 3.3. Analytical Sensitivity

The limit of detection in terms of DNA copy numbers was determined using 10-fold serial dilutions of a cloned 933 bp segment of N of CVS-11 containing 10^6^ to 10^0^ DNA copies/PCR reaction. In the hnPCR, the limit of detection was 100 DNA copies/3 *μ*L of starting material of the CVS-11 plasmid. In the real-time PCR (NDWD), the 10-fold serial dilution of the CVS-plasmid was tested in triplicate. The limit of detection was 10 DNA copies/2 *μ*L of the CVS-11 plasmid with CT-values between 38.6 and 47.5. The efficiency was at 82.8% with a slope coefficient of −3.82 (not shown). The intra-assay repeatability of the real-time PCR (NDWD) proved to be excellent with very low coefficients of variation up to 10^2^ DNA copies (0.18–1.37%) with and outlier at 10^1^ copies (12.05%; Table 3).

### 3.4. Diagnostic Broadness of PCR Methods

**Figure 3:**
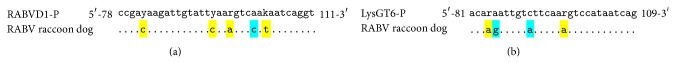
Alignment of probes RABVD1-P (a) and LysGT6-P (b) with a RABV strain isolated from a raccoon dog from Poland. Matches in nondegenerated positions are displayed as dots. Matches in wobbled positions are highlighted in yellow. Mismatches are highlighted in turquoise.

A total of 31 lyssaviruses (19 identified as classical RABV, 7 as EBLV-1, 4 as EBLV-2, and 1 as DUVV) were used to test the diagnostic performance of the PCR (Table 1). All viruses used were detected with the expected band size in the hnRT-PCR technique (Table 4). The specificity of the amplified product was confirmed as RABV, EBLV-1, EBLV-2, or DUVV by sequencing in each case. Host GAPDH (glyceraldehyde 3-phosphate dehydrogenase) was detected in all of the cell culture supernatants and mouse brain suspensions as internal control, indicating that sample material was present and RNA isolation, reverse transcription, and amplification were not inhibited. All samples were also tested positive by the real-time RT-PCR assay (Table 4). Sendai virus as internal control for inhibition was run in parallel using the same cycling conditions. In the real-time RT-PCR (NDWD) with an annealing temperature of 50°C, some samples were detected with more than one probe. With the exception of the sample derived from a raccoon dog from Poland, the probe for the homologous genotype always yielded the lowest CT-value. In this exceptional case, the probe for the detection of genotype 6 was slightly lower than the one for genotype 1 (15.0 versus 16.1; Table 4). Comparison of these two probes with the sequence of the rabies variant in question showed 1 and 2 mismatches with the RABVD1-P and LysGT6-P probe, respectively (Figure 3). Duvenhage virus, also designated as genotype 4, was detectable with the probe adapted for genotype 6 in spite of 3 mismatches (LysGT6-P). The probe LysGT6-P was able to detect synthetic DNA fragments of BBLV, ARAV, and IRKV, although containing one or two mismatches, respectively. The synthetic DNA fragments of KHUV, ABLV, LBV, MOKV, SHIBV, and WCBV, which exhibited from four (ABLV/KHUV) up to ten (WCBV) mismatches to the best fitting probe for GT1, GT6, or GT5, respectively, were all detected with the corresponding species-specific new probe (Table 2). 10^5.2^ copies of the synthetic DNA were detected at CT-values of 27.9–33.5 for ABLV, MOKV, SHIBV, and WCBV (not shown). The efficiency of detection of KHUV, which showed an atypical amplification plot at the highest number of 10^10.2^ copies of the synthetic DNA, and that of LBV was lower with CT-values of 40.0 and 40.5 at a copy number of 10^5.2^, respectively (not shown). Multiplexing of combinations of these probes (ivvLBV-P (FAM: 6-carboxyfluorescein), ivvKhujand-P (YY: Yakima Yellow), and ivvSBLV2-P (Cy5: Cyanine 5), as well as ivvWCBV-P (FAM), ivvMok-P (YY), and ivvABLV2n-P (Cy5)) as for the species 1, 5, and 6 was not successful.

### 3.5. Sequencing of Amplification Products and Phylogenetic Analysis

**Figure 4:**
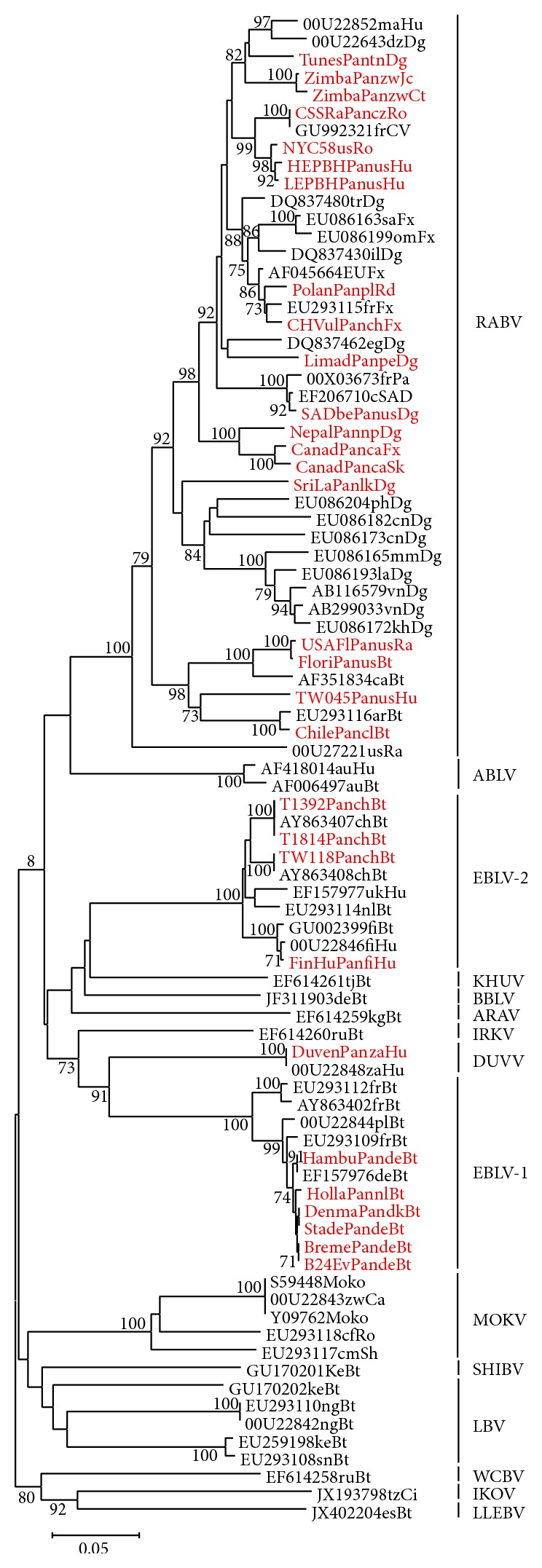
Phylogenetic tree with 543 bp fragments of N. Phylogenetic tree obtained with MEGA5 software [35]. The length of branches (horizontal lines) corresponds to phylogenetic distance between different sequences (scale bar in substitutions per site). Numbers proximal to nodes indicate bootstrap confidence of subjacent groups. Lyssavirus strains used in this study are depicted in red. Sequences are given with GenBank or own designation followed by the two-letter country code and a two-letter code for the source species as follows: Bt, bat; Ca, cat; Ci, African civet; Dg, dog; Fx, fox; Hu, human; Jc, jackal; Ra, raccoon; Rd, raccoon dog; Ro, rodent; Sh, shrew; Sk, skunk, except for Pa, cSAD, and CV, which are standing for Pasteur strain, Street Alabama Dufferin strain, and challenge virus standard, respectively. The currently used abbreviations for species are shown next to the tree. RABV, rabies virus; ABLV, Australian bat lyssavirus; WCBV, West Caucasian bat virus; IKOV, Ikoma virus; LLEBV, Lleida bat lyssavirus; MOKV, Mokola virus; SHIBV, Shimoni bat virus; LBV, Lagos bat virus; KHUV, Khujand virus; BBLV, Bokeloh bat lyssavirus; ARAV, Aravan virus; DUVV, Duvenhage virus; EBLV, European bat lyssavirus; IRKV, Irkut virus.

The 543 bp amplification products of the nucleoprotein gene obtained in the heminested PCR reactions were sequenced (Table 1) and analysed phylogenetically with additional sequences retrieved from GenBank representing all known lyssaviruses. The similarity of the 543 bp nucleotide sequences of the nucleoprotein gene (positions 74–616 according to the Pasteur virus genome) among the lyssaviruses included ranged from 64.6% to 90.1% (Hamming distance). Similarity at the amino acid level was 68.0–95%. The resulting phylogenetic tree obtained by the neighbor-joining method implemented in the MEGA5 software is presented in Figure 4. All known genotypes were resolved with high bootstrap confidence with our samples grouping expectedly.

### 3.6. Clinical Specimens

Clinical specimens like brain suspensions (1x human, 12x mouse), skin biopsies from the nape of the neck (3x human, 1x mouse), saliva (4x human), and cerebrospinal fluids (6x human) were used for the establishment of the assays. Both hnRT-PCR and real-time RT-PCR (NDWD) were shown to work properly on all these samples without significant inhibition using GAPDH as internal control for conventional RT-PCR and Sendai virus for real-time RT-PCR (NDWD), which was mixed to the samples before RNA isolation. Skin biopsies taken from the neck and lip of a mouse (white Swiss mouse at an age of 3 weeks) euthanized 2 weeks after intracerebral infection with SAD Bern virus 20 years ago were positive with the hnRT-PCR and real-time PCR (NDWD). Skin biopsies taken from other locations on the head were positive with the real-time PCR (NDWD) and weakly positive in hnRT-PCR whereas samples taken around the vibrissae were weakly positive in real-time PCR (NDWD) only. Sequencing of amplification products excluded a contamination with the positive CVS-11 control (not shown).

Brain suspension from an imported human rabies case in 2012 was already strongly positive after one round of the hnRT-PCR. Phylogenetic analysis of the 543 bp nucleotide fragment revealed its close relationship to the classical rabies virus strains circulating in the insectivorous Mexican free-tailed bat,* Tadarida brasiliensis*, a species common in the southern United States and Mexico. This allowed identification of the origin of the patient's infection, who had travelled extensively and did not report any previous biting incident [51, 52].

## 4. Discussion

Based on a large amount of published work, we were able to establish and quantitatively characterise RT-PCR protocols for the detection of lyssaviruses in clinical samples. Well suited real-time protocols [8, 25] for the detection of classical rabies virus and the genotypes 5 and 6 (EBLV-1 and EBLV-2) were adapted and extended for the detection of at least 13 lyssavirus species. To this goal, 6 species-specific probes (KHUV, ABLV, LBV, MOKV, SHIBV, and WCBV) were designed and verified on synthetic DNA fragments encompassing the targeted sequence of the lyssaviral nucleoprotein gene. Multiplexing the probes in a single-tube reaction as described for the genotypes 1, 5, and 6 was not possible with the newly designed probes, probably due to false priming and/or interference within the mix of reagents and synthetic target sequence [53]. As far as evaluated with the multiplexed probes for the genotypes 1, 5, and 6 using 31 viral isolates, which were all detectable with high sensitivity, direct differentiation of targeted genotypes on the base of the quantitative reaction (CT-values) was mostly possible. Furthermore, a single probe for genotype 6 (species EBLV-2) was able to detect up to 7 genotypes/species. We consider this type of cross hybridization as an advantage of the technique using degenerate primers and probes in terms of a broad detectability of lyssavirus rather than a lack of accuracy of discrimination. In an approach using SYBR Green qPCR with similarly degenerate primers spanning the same part of N as in this work [28], the detection of all lyssavirus species known at that time was achieved. Using several genotype-specific probes in a TaqMan real-time protocol, we were able to add more intrinsic confidence to the specificity of the analysis [54]. Quantitative characterisation of the assay using the probe for genotypes 1, 5, and 6 on CVS-11 showed excellent sensitivity and repeatability with a detection limit of as low as an infectious dose of 0.003 FFD_50_ combined with an analytical sensitivity of 10 DNA copies at efficiencies of 94.5% and 82.8%, respectively. Considering the amount of degeneration in both primers and probes, the efficiencies determined must be considered satisfactory. As far as the absolute detection limit in terms of target copies is concerned, the usage of plasmid DNA rather than RNA must be kept in mind. Since reverse transcription cannot be assumed as 100% efficient and reproducible [55], the limit for RNA might be somewhat lower.

For further characterisation of samples with a positive reaction in real-time RT-PCR, excellent simple and (hemi)nested RT-PCR protocols are available. With the protocol used for this work [22], all 31 viral strains used could be amplified and sequenced, confirming the diagnostic broadness of the assay. Phylogenetic analysis of these partial nucleotide sequences belonging to genotypes 1, 4, 5, and 6 along with known sequences confirmed the suitability of this relatively conserved genomic region for molecular-epidemiological characterisation of lyssaviruses circulating worldwide [56–58]. This was also confirmed in the context of a human rabies case imported to Switzerland in 2012, which could be attributed unequivocally to an exposure to a bat of the species* Tadarida brasiliensis* in California, USA.

## 5. Conclusion

In this work we could show and validate the suitability of an adapted and further developed real-time RT-PCR protocol for the rapid, efficient, and highly sensitive intravitam diagnosis of a wide spectrum of lyssavirus species followed by rapid molecular-epidemiological characterisation of viral isolates respective to origin of the virus and source of exposure, using heminested RT-PCR. This new tool is also particularly promising for active surveillance of European bat lyssaviruses using oral swabs in live-captured bats.

## Figures and Tables

**Table 1:** Rabies viruses used.

Species	Designation	Host species	Origin, year of isolation/receipt^a^	Material	Accession number^b^
EBLV-1	Bat Stade	Bat (*E. serotinus*)	Germany (Stade), 1970	Mouse brain suspension	KF831524/KF831550
EBLV-1	Bat Hamburg RV 9	Bat (*E. serotinus*)	Germany (Hamburg), 1968	Mouse brain suspension	KF831526/KF831552
EBLV-2	Bat Finland RV 8	Human, bat exposure	Finland, 1986	Mouse brain suspension	KF831528/KF831553
EBLV-1	Bat Holland RV 31	Bat	Netherlands, 1988^a^	Mouse brain suspension	KF831525/KF831551
RABV	Dog Tunisia	Dog	Tunisia, 1986	BHK-21 cell culture supernatant	KF831519/KF831545
EBLV-1	Bat Spain RV 119	Bat	Spain, 1989^a^	BHK-21 cell culture supernatant	KF831527
RABV	Bat Florida	Yellow bat	USA (Florida), 1986^a^	BHK-21 cell culture supernatant	KF831522/KF831548
EBLV-1	Bat Denmark	Bat (*E. serotinus*)	Denmark, 1986^a^	BHK-21 cell culture supernatant	KF831523/KF831549
RABV	Raccoon dog Poland	Raccoon dog	Poland, 1985	Mouse brain suspension	KF831518/KF831544
RABV	Raccoon Florida	Raccoon	USA (Florida), 1986	BHK-21 cell culture supernatant	KF831521/KF831547
RABV	Bat Chile RV 108	Bat	Chile, 1988^a^	Mouse brain suspension	KF831520/KF831546
RABV	Jackal Zimbabwe	Jackal	Zimbabwe, 1990^a^	Mouse brain suspension	KF831529/KF831554
RABV	Cattle Zimbabwe	Cattle	Zimbabwe, 1990^a^	Mouse brain suspension	KF831555
RABV	Canada Red Fox	Red Fox	Canada (Ontario), 1986^a^	Mouse brain suspension	KF831530/KF831556
RABV	LEP	Human	USA (Georgia), 1939	BHK-21 cell culture supernatant	KF831531/KF831557
RABV	HEP	Human	USA, 1939	BHK-21 cell culture supernatant	KF831532/KF831568
RABV	Dog Nepal Nr. 96	Dog	Nepal, 1989^a^	BHK-21 cell culture supernatant	KF831534/KF831559
RABV	Sri Lanka dog 121	Dog	Sri Lanka, 1986	BHK-21 cell culture supernatant	KF831535/KF831572
RABV	Eurofox 912/87	Fox	Switzerland, 1987	BHK-21 cell culture supernatant	KF831538/KF831562
DUVV	Duvenhage RV6	Human	South Africa, 1988^a^	BHK-21 cell culture supernatant	KF831533/KF831558
RABV	NYC 58	Laboratory strain	USA, 1987^a^	Mouse brain suspension	KF831539/KF831563
RABV	Dog Lima	Dog	Peru (Lima), 1985^a^	Mouse brain suspension	KF831540/KF831564
EBLV-1	Bat Bremerhaven RV11	Bat	Germany (Bremerhaven), 1989^a^	BHK-21 cell culture supernatant	KF831541/KF831565
EBLV-1	Bat B24	Bat	Europe, 1986^a^	Mouse brain suspension	KF831536/KF831560
RABV	Skunk Canada	Striped skunk	Canada (Ontario), 1986	BHK-21 cell culture supernatant	KF831537/KF831561
RABV	SAD Bern	ERA∗	USA, 1935 (received 1976)	BHK-21 cell culture supernatant	KF831542/KF831566
RABV	CSSR/A-virus	Rodent, laboratory strain	CSSR (Prague), 1987^a^	BHK-21 cell culture supernatant	KF831543/KF831567
RABV	Challenge virus standard (CVS-11) ATCC VR 959	Laboratory strain	France (CNEVA), 1995^a^	BHK-21 cell culture supernatant	GU992321
EBLV-2	TW 1814/92	Bat (*M. daubentonii*)	Switzerland (Plaffeien), 1992	Na 42/13 cell culture supernatant	KF831569
EBLV-2	TW 1392/93	Bat (*M. daubentonii*)	Switzerland (Versoix), 1993	Na 42/13 cell culture supernatant	KF831570
EBLV-2	TW 118/02	Bat (*M. daubentonii*)	Switzerland (Geneva), 2002	Na 42/13 cell culture supernatant	KF831571

Lyssaviruses of 4 different species were tested.

^a^Year of receipt at the Swiss rabies center.

^b^Accession numbers of 220 bp and 543 bp N fragments, respectively, sequenced in this work.

∗Reference [36].

**Table 2:** Primers and probes.

Method	Name	Sequence	Length	Position^a^	Product	Reference
hnRT-PCR	JW12-F	ATGTAACACCYCTACAATG	19	55–73		Panning et al., 2010 [22]
	JW6AS1-R1	CAATTGGCACACATTTTGTG	20	660–641	606 bp	
	JW6AS2-R1	CAGTTAGCGCACATCTTATG	20	660–641	606 bp	
	JW10AS1-R2	GTCATCAATGTGTGATGTTC	20	636–617	582 bp	
	JW10AS2-R2	GTCATTAGAGTATGGTGCTC	20	636–617	582 bp	

Cloning RT-PCR	TWclon-F	ACGCTTAACRACMAAACCAG	20	1–20		This work
	TWclon-R	TGKATGAARTAAGAGTGWGGRAC	23	933–911	933 bp	

Control^1^	GAPDH-F	GGCAAGTTCCATGGCACAGT	20	58–72^b^		Ravazzolo et al., 2006 [37]
	GAPDH-R	ACGTACTCAGCACCAGCATCAC	22	161–182^b^	125 bp	

Real-time RT-PCR	RABVD1-F	ATGTAACACCYCTACAATG	19	55–73		Nadin-Davis et al., 2009 [8]
	RABVD1-R	GCMGGRTAYTTRTAYTCATA	20	165–146	111 bp	Nadin-Davis et al., 2009 [8]
	RABVD1-P	5′-FAM-CCGAYAAGATTGTATTYAARGTCAAKAATCAGGT-BHQ1-3′	34	78–111		Nadin-Davis et al., 2009 [8]
	LysGT5-P	5′-YY-AACARGGTTGTTTTYAAGGTCCATAA-BHQ1-3′	26	80–105		Wakeley et al., 2005 [25]
	LysGT6-P	5′-Cy5-ACARAATTGTCTTCAARGTCCATAATCAG-BHQ2-3′	29	81–109		Wakeley et al., 2005 [25]
	ivvWCBV-P	5′-FAM-TCGGATATCACTTCGGGTTTGAGAGTCA-BHQ1-3′	28	141–114		This work
	ivvMok-P	5′-YY-TTGTGTTCAAGGTGAAYAAYCAAGT-BHQ1-3′	25	87–111		This work
	ivvABLV2n-P	5′Cy5-ATTGTCTTTAAGGTCAACAATCAGTT-BHQ2-3′	26	86–111		This work
	ivvLBV-P	5′-FAM-ATTGTTTTCAAAGTYCAYAATCAGGTMGTGTC-BHQ1-3′	32	86–117		This work
	ivvKhujand-P	5′-YY-ACAGAATTGTCTTYAAAGTYMAKAATCA-BHQ1-3′	28	81–108		This work
	ivvSBLV2-P	5′-Cy5-TCWGAGATTATRTCTGGCTTCAAAGACAC-BHQ2-3′	29	141–113		This work

Control^1^	Sendai-F	GTCATGGATGGGCAGGAGTC	20	8553–8572^b^		Kaiser, 2001 [38]
	Sendai-R	CGTTGAAGAGCCTTACCCAGA	21	8788–8768^b^	236 bp	
	Sendai-P	5′-FAM-CAAAATTAGGAACGGAGGATTGTCCCCTC-Tamra-3′	29	8720–8748^b^		

Changed bases referring to the reference are underlined. Wobbled positions are as follows: Y = C/T, X/N = G/A/T/C, W = A/T, S = C/G, R = A/G, M = A/C, K = G/T, H = A/C/T, and B = C/G/T.

^a^Rabies primer and probe positions are given according to the Pasteur virus genome (accession number X03673).

^b^Positions of the GAPDH-primers refer to the GenBank sequence AJ431207; positions of the Sendai-primers refer to the GenBank sequence M30202.

^1^Controls for classical and real-time RT-PCR.

FAM: 6-carboxyfluorescein reporter dye, BHQ-1: Black Hole Quencher-1, YY: Yakima Yellow, Cy5: Cyanine 5, BHQ-2: Black Hole Quencher-2, and Tamra: carboxy-tetramethyl-rhodamine.

**Table 3:** Serial dilutions of the CVS-11 plasmid in real-time PCR (NDWD).

Dilution	Copy numbers/2 *μ*L	CT-value 1	CT-value 2	CT-value 3	Mean ± SD	CV (%)
10^−1^	10^6^	22.6	22.0	22.4	22.3 ± 0.31	1.37
10^−2^	10^5^	25.1	25.2	25.3	25.2 ± 0.10	0.40
10^−3^	10^4^	29.1	28.8	28.7	28.9 ± 0.21	0.72
10^−4^	10^3^	32.9	32.9	32.8	32.9 ± 0.06	0.18
10^−5^	10^2^	36.4	36.2	35.9	36.2 ± 0.25	0.70
10^−6^	10^1^	47.5	38.6	39.0	41.7 ± 5.03	12.05
10^−7^	10^0^	—	—	—	—	—

SD: standard deviation; CV: coefficient of variation.

**Table 4:** PCR-results of rabies viruses tested.

					Real-time RT-PCR (NDWD)	
Sample		Species	hnRT-PCR		CT-value^a^	
				FAM (GT1)	YY (GT5)	Cy5 (GT6)
1	Bat Stade (1970)	EBLV-1	+		14.7	17.3
2	Bat Hamburg RV 9 (1968)	EBLV-1	+	20.2	14.8	16.4
3	Bat Finland RV 8 (1986)	EBLV-2	+			15.1
4	Bat Holland RV 31	EBLV-1	+	20.2	14.7	16.8
5	Dog Tunisia (1986)	RABV	+	24.6	27.2	33.2
6	Bat Spain RV 119	EBLV-1	+	33.4	27.6	31.6
7	Bat Florida	RABV	+	24.2	41.6	
8	Bat Denmark	EBLV-1	+	26.5	21.1	22.4
9	Raccoon dog Poland (1985)	RABV	+	16.1		15.0
10	Raccoon Florida (1986)	RABV	+	25.8		
11	Bat Chile RV 108	RABV	+	14.4		
12	Jackal Zimbabwe	RABV	+	14.6		
13	Cattle Zimbabwe	RABV	+	18.0		
14	Canada Red Fox	RABV	+	17.9		
15	LEP (Flury, 1939)	RABV	+	17.7		
16	HEP (Flury)	RABV	+	28.5		
17	Dog Nepal Nr. 96	RABV	+	21.7		
18	Sri Lanka dog 121 (1986)	RABV	+	23.2		
19	Eurofox 912/87	RABV	+	28.3		
20	Duvenhage RV6	DUVV	+			27.0
21	NYC 58	RABV	+	16.5		
22	Dog Lima	RABV	+	20.0		
23	Bat Bremerhaven RV11	EBLV-1	+		27.0	
24	Bat B24	EBLV-1	+		16.9	20.6
25	Skunk Canada	RABV	+	23.2		
26	SAD Bern, 1935	RABV	+	18.8		
27	CSSR/A-virus	RABV	+	18.5		
28	Challenge virus standard (CVS-11) ATCC VR 959	RABV	+	19.8		
29	TW 1814/92	EBLV-2	+
30	TW 1392/93	EBLV-2	+
31	TW 118/02	EBLV-2	+

^a^Only for samples with positive reaction.

FAM: 6-carboxyfluorescein reporter dye, to detect RABV (GT1); YY: Yakima Yellow, to detect EBLV-1 (GT5); Cy5: Cyanine 5, to detect EBLV-2 (GT6); ND: not done.
